# Biobran/MGN-3, an Arabinoxylan Rice Bran, Exerts Anti-COVID-19 Effects and Boosts Immunity in Human Subjects

**DOI:** 10.3390/nu16060881

**Published:** 2024-03-19

**Authors:** Sudhanshu Agrawal, Anshu Agrawal, Mamdooh Ghoneum

**Affiliations:** 1 Division of Basic and Clinical Immunology, Department of Medicine, University of California, Irvine, Irvine, CA 92697, USA; sagrawal@uci.edu (S.A.); aagrawal@uci.edu (A.A.); 2 Department of Surgery, Charles R. Drew University of Medicine and Science, Los Angeles, CA 90059, USA; 3 Department of Surgery, University of California, Los Angeles, Los Angeles, CA 90095, USA

**Keywords:** Biobran/MGN-3, anti-COVID-19, SARS-CoV-2, DCs, monocytes, IFN-α, CD8 T cells

## Abstract

Corona Virus Disease 19 (COVID-19) has been a major pandemic impacting a huge population worldwide, and it continues to present serious health threats, necessitating the development of novel protective nutraceuticals. Biobran/MGN-3, an arabinoxylan rice bran, is a potent immunomodulator for both humans and animals that has recently been demonstrated to protect against severe acute respiratory syndrome coronavirus 2 (SARS-CoV-2) in vitro. We here investigate Biobran/MGN-3′s potential to enhance an antiviral immune response in humans. Peripheral blood mononuclear cells (PBMCs) derived from eight subjects taking Biobran/MGN-3 (age 55–65 years) and eight age-matched control subjects were stimulated with irradiated SARS-CoV-2 virus and then subjected to immuno-phenotyping and multiplex cytokine/chemokine assays. Results showed that PBMCs from subjects supplemented with Biobran/MGN-3 had significantly increased activation of plasmacytoid dendritic cells (pDCs) coupled with increased IFN-α secretion. We also observed higher baseline expression of HLA-DR (human leukocyte antigen-DR isotype) on dendritic cells (DCs) and increased secretion of chemokines and cytokines, as well as a substantial increase in cytotoxic T cell generation for subjects taking Biobran/MGN-3. Our results suggest that Biobran/MGN-3 primes immunity and therefore may be used for boosting immune responses against SARS-CoV-2 infections and other diseases, particularly in high-risk populations such as the elderly.

## 1. Introduction

The COVID-19 pandemic caused by severe acute respiratory syndrome coronavirus 2 (SARS-CoV-2) is ongoing and poses a continuing threat to populations worldwide, generating a wide range of symptoms and diseases [1,2]. The SARS-CoV-2 virus commonly causes influenza-like symptoms (lethargy, fever, cough) and may even go unnoticed. Nevertheless, in certain high-risk populations, the disease can run a severe course, even causing multiple organ failure and death. Emerging data indicate that severe COVID-19 is linked to immune system dysregulation, particularly for CD8 T cell function. IFN-γ and TNF-α production are impaired, and PD-1 expression is upregulated [3]. Since naïve T cells are primed by dendritic cells (DCs), it is thought that changes in the function of DCs may lead to the reduced CD8 T cell priming found in patients with severe COVID-19. Therefore, the severity of COVID-19 may be a consequence of impaired innate immunity [4,5]. DCs and macrophages are key cellular components of the innate immune system, as they can recognize viral threats and respond by becoming activated and producing inflammatory mediators that alert and prime other cells to the threat [6,7]. DCs produce interferons (IFNs) of type I and III, among other cytokines, that enhance the antiviral activity of cytotoxic CD8 T cells [8,9,10]. Given the pivotal roles of monocytes and DCs in initiating host defenses against viral infections, agents that enhance their functions may be beneficial for fighting COVID-19.

Beyond vaccines, there are few preventative options available to groups with the highest risk for severe COVID-19, such as elderly individuals, who experience a broad decline in immune function [11]. Therefore, it is worthwhile to investigate the potential of certain biological response modifiers (BRMs) to boost the antiviral immune response and lower hospitalization risk. There have been earlier studies over an extensive range of natural products, herbal supplements, and traditional medicine products that have been reported to work against COVID-19 in vitro [12,13,14,15,16,17,18,19,20,21,22,23,24,25,26,27,28]. However, we are not aware of an in vivo human study that was carried out showing the efficiency of a food supplement in exerting anti-COVID-19 effects, though vitamin C has been shown to shorten patient stays in intensive care units by 8.6% [29] and to shorten the period under mechanical ventilation by 18.2% [30].

Among the natural products, Biobran/MGN-3, an arabinoxylan rice bran, has recently been demonstrated to protect against SARS-CoV-2 in vitro [31]. An important objective of the current study is to clarify our understanding of how Biobran/MGN-3 modulates the initial activation of monocytes and DCs following SARS-CoV-2 infection. Biobran/MGN-3 has been shown to have a significant ability to modulate various arms of the immune response in vitro [32]. It activates dendritic cells and CD4 cells; it increases secretion of type I IFNs, type III IFNs, and IFN-γ [33,34]; and it enhances the phagocytic activity of macrophages [35]. Additionally, it activates natural killer (NK) cells [36] and increases human B and T cell mitogen responses. Remarkably, Biobran/MGN-3 supplementation has also been found to reduce the incidence of influenza-like illnesses in elderly subjects [37], act against HIV in vitro [38], and significantly reduce the viral load of patients infected with chronic hepatitis C [39]. Biobran/MGN-3 thus has the capacity to enhance antiviral responses. Furthermore, Biobran/MGN-3 has been shown to be a potent anticancer agent. Biobran/MGN-3-stimulated DCs were able to prime cytotoxic CD8 cells and increase cancer-killing activity [33,34], and Biobran/MGN-3 has been demonstrated to modulate psychoneuroimmune activity and thereby enhance the health-related quality of life in healthy older adults [40] and in cancer patients [36,37].

Motivated by the above results, our hypothesis was that Biobran/MGN-3 could induce antiviral activity against SARS-CoV-2. In the present work, eight healthy subjects taking Biobran/MGN-3 as a supplement were age-matched against eight healthy controls. The primary hypothesis was that subjects orally taking Biobran/MGN-3 as a supplement will exhibit better innate immune system activation against viral infection compared to normal age-matched healthy donors. To investigate this, we used our previously established method of studying innate immune activation using irradiated SARS-CoV-2 [41]. Using this method, we study the early activation of DCs and monocytes 24 h after infection. This is different from other studies in which the activation of these cells is studied five to six days after infection when the patient has already become symptomatic and presents to the clinic. In this case, the period of secondary activation following release of cytokines and other inflammatory mediators [42] and the initial activation of monocytes and DCs is missed. In the present work, we planned to determine Biobran/MGN-3′s impact on the initial activation of DCs and monocytes and the effect this has on downstream CD8 T cell responses.

## 2. Materials and Methods

### 2.1. Blood Donors

Samples of peripheral blood were obtained from healthy volunteers, 55–65 years old, taking Biobran/MGN-3 (1–2 gm/day for one year) and from control subjects with the help of the Institute for Clinical and Translational Immunology (ICTS) and nurses at the Gottschalk clinic at UC Irvine. The sample volunteers for the Biobran/MGN-3 group were chosen subject to the constraint that they had been taking Biobran/MGN-3 at a typical recommended supplement dose (1–2 gm/day) for at least one year—the dose was not explicitly specified beforehand. Blood was drawn under a protocol approved by the University of California Irvine’s Institutional Review Board. Written, informed consent was obtained from the subjects. A total of 20 mL of blood was collected into BD Vacutainer tubes with sodium heparin. Fresh PBMCs were used for the experiment.

### 2.2. Biobran

Biobran is a denatured hemicellulose that is extracted from rice bran by reacting rice bran hemicellulose with carbohydrate-hydrolyzing enzymes obtained from Shiitake mushrooms. Biobran’s main chemical structure consists of arabinoxylan with an arabinose polymer in its side chain and xylose in its main chain [31]. Biobran was being taken by the subjects in its commercially available form.

### 2.3. Antibodies and Reagents

Cells were stained with the following antibody clones:

DCs: CD14 BV650 (cloneM5E2), CD86 (clone IT2.2), CD123 BV421 (clone 6H6), CD11c APC (cloneBu15), HLADR PerCP (clone L-243), and lineage FITC were obtained from BioLegend (San Diego, CA, USA). Live/dead fixable viability stain 510 was obtained from BD Pharmingen (San Jose, CA, USA).

CD8 T: Granzyme B AL647 (cloneGB11), CD107aPE (clone H4A3), Perforin FITC (cloneB-D48), and CD8 PerCP (clone-SK1) were obtained from BioLegend.

Virus: Control irradiated Vero cell lysate and heat-inactivated and irradiated SARS-CoV-2 isolate USA-WA1/2020 were obtained from BEI resources (NIAID). Gamma-irradiation was performed as per the BEI resources using 5 × 10^6^ RADs on dry ice; heat inactivation was accomplished by heating for 30 min at 65 °C. The inactivated viruses are biosafety level 1.

Peripheral blood mononuclear cells (PBMCs): PBMCs were isolated from donors’ blood by the density gradient centrifugation method using a lymphocyte separation medium (Fisher Scientific, Pittsburgh, PA, USA). Briefly, 20 mL of blood was diluted with 10 mL of HBSS, gently overlaid to 15 mL with lymphocyte separation media in a 50 mL tube, and centrifuged for 15 min at 2000 rpm. The monolayer was collected and washed, and cells were counted. A total of 10 µg of irradiated SARS-CoV-2 virus were used to stimulate 10^6^ PBMC/mL in RPMI 1640 containing 10% fetal bovine serum (Gibco, Thermo Fisher Scientific, Pittsburgh, PA, USA) as described previously v [41]. After 24 h, half of the cells and supernatants were collected. Specific antibodies were used to stain cells for activation of monocytes and DCs. Supernatants were stored at −70 °C for quantification of innate chemokines and cytokines. The remaining cells were left in culture for another six days, following which cells were gathered and stained for cytotoxic T lymphocytes. Supernatants were stored at −70 °C for quantification of adaptive immune cytokines.

### 2.4. Immuno-Phenotyping (Flow Cytometry)

Following 24 h of stimulation, PBMCs were collected and stained as per the manufacturer’s protocol with fixable viability stain 510 for live/dead cell exclusion. Afterwards, cells were washed and surface stained with specific antibodies for monocytes and DCs for 30 min in the dark at RT. Cells were then washed and fixed using 2% PFA. FMOs and isotype controls were used. Acquisition was carried out by a BD FACS Celesta equipped with a BVR laser (BD Biosciences, San Jose, CA, USA). Side and forward scatters and singlets were used to gate and exclude cellular debris. A total of 3 × 10^4^ cells were acquired/sample. FlowJo software v10.10 (Ashland, OR, USA) was used for analysis. DC identification was performed by the following phenotypes: monocytes—CD14+/HLA−DR+; pDC—Lin−/HLA−DR+/CD123+; mDCs—Lin−/HLA−DR+/CD11c+. The gated populations were used to determine CD86 expression.

For staining of cytotoxic CD8 T cells, cells collected at day 7 were stained with fixable viability stain 510 for live/dead cell exclusion. Cells were then washed, and CD8 antibody was used for surface staining for 30 min, following which cells were fixed, made permeable by fix perm buffer (BD Biosciences), and intracellularly stained with granzyme B and perforin. Appropriate FMOs and isotype controls were used. FlowJo (BD Biosciences) was used for analysis.

### 2.5. Multiplex Cytokine/Chemokine Assay

Multiplex kits (Thermo Fisher Scientific, USA) were used for the assay of culture supernatants collected at day 1 and at day 7 after stimulation. The kit used for day 1 supernatants contained the analytes CCL-19, CXCL-10, IFN-α, TNF-α, CCL-2, IL-29, IL-8, and IL-6. The kit for day 7 supernatants contained the analytes IFN-α, IL-6, IFN-γ, and granzyme B. The manufacturer’s protocol was followed according to procedure. Briefly, supernatants were mixed overnight with premixed beads (30 cytokines). Following incubation with detection antibodies and Streptavidin-PE for 1 h each, plates were run on Magpix for the identification of specific cytokines.

### 2.6. Statistical Analysis

GraphPad Prism version 10 (GraphPad Inc., San Diego, CA, USA) was used for statistical analysis. Comparisons between two groups were performed using paired or unpaired *t* tests. The analysis of two or more groups was performed using a one way ANOVA followed by Tukey’s multiple comparison test. A *p* < 0.05 was determined to be statistically significant. Figure legends state the specific statistical considerations for each result.

## 3. Results

### 3.1. DCs and Monocytes Are Activated by SARS-CoV-2 in PBMCs

**Figure 1:**
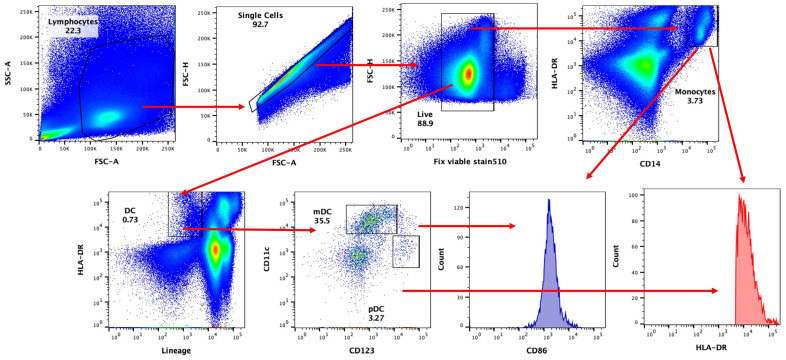
Gating strategy for DCs and monocytes. PBMCs were stimulated O/N, and flow cytometry was performed. Cells were stained for live cells for pDCs, mDCs, and monocyte subsets. Co-stimulation markers CD86 and HLA-DR were checked in these cells. Gated live cells were checked for singlet population. These gated cells were gated for lineage HLA-DR + (DCs). DCs were further gated for CD11c (mDCs), CD123 (pDC), and CD14 (monocytes). Mean fluorescent intensity (MFI) for CD86 and HLADR was calculated in mDCs, pDCs, and monocytes.

The response of monocytes and DCs to irradiated SARS-CoV-2 virus in subjects taking Biobran/MGN-3 as a supplement was compared to age-matched control subjects. The gating strategy used for identifying mDCs (myeloid DCs), pDCs (plasmacytoid DCs), and monocytes is shown in Figure 1.

**Figure 2:**
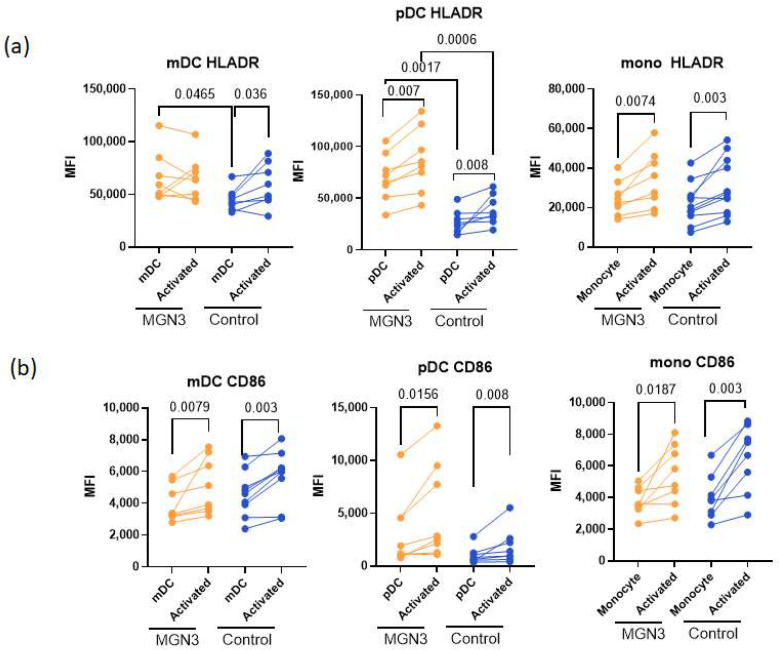
DCs and monocytes from Biobran/MGN-3-supplemented subjects display enhanced activation in response to SARS-CoV-2 compared to age-matched controls. Lines connect the unactivated and activated conditions in the same subject. (**a**) HLA-DR expression on gated pDCs (lineage− HLADR+CD123+), gated mDCs (lineage− HLADR+CD11c+), and gated CD14+ monocytes. (**b**) CD86 expression on gated pDCs, mDCs, and monocytes. *N* = 8 for both Biobran/MGN-3-supplemented subjects and for age-matched controls. The *p*-values between the unactivated control and SARS-CoV-2-activated conditions in Biobran/MGN-3 and control subjects were calculated using a paired *t*-test (parametric).

Figure 2a indicates that HLA-DR was significantly upregulated upon SARS-CoV-2 stimulation for both Biobran/MGN-3-supplemented and control groups for pDCs and monocytes but not in mDCs. The HLA-DR upregulation was significantly higher for pDCs in the group supplemented with Biobran/MGN-3 than for the controls, indicating increased activation. In contrast, monocytes from both groups displayed similar levels of HLA-DR upregulation, while mDCs displayed significant upregulation. Remarkably, both mDCs and pDCs displayed increased expression of HLA-DR at baseline for the Biobran/MGN-3 group relative to control.

The expression of another activator marker, CD86, was significantly upregulated on SARS-CoV-2 stimulation in both Biobran/MGN-3-supplemented and control groups for all three cell types, with no significant difference in activation between groups (Figure 2b). The expression was also similar at baseline. Taken together, these results indicate that MGN-3 primes the mDCs and pDCs, inducing a semi-activated state, as evidenced by increased HLA-DR expression at baseline.

### 3.2. Secretion of Soluble Mediators from SARS-CoV-2-Stimulated PBMCs at 24 h

**Figure 3:**
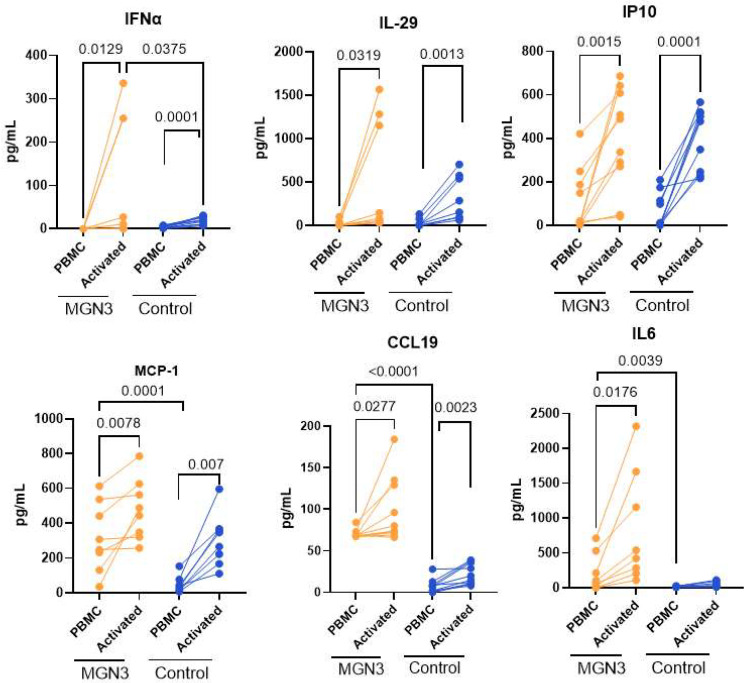
Differential cytokine/chemokine secretion in Biobran/MGN-3-supplemented subjects versus age-matched controls in response to SARS-CoV-2. Plots show quantification of cytokines/chemokines in the supernatant by multiplex. Lines connect the unactivated and activated conditions in the same subject. *N* = 8 for both Biobran/MGN-3-supplemented subjects and for age-matched controls. A Wilcoxon matched pairs signed-rank test was used to calculate *p*-values between the control and SARS-CoV-2-activated conditions in males and females. The unpaired *t*-test (Mann–Whitney test) was used to assess the significance between Biobran/MGN-3 and the control.

The secretion of interferons (IFNs) and other cytokines/chemokines by DCs and monocytes early in the immune response is a very important measure of antiviral activity. Therefore, cytokines and chemokines were measured after 24 h of viral stimulation. IFN-α (Type I IFN) and IL-29 (Type III IFN) were significantly induced in both the Biobran/MGN-3 and age-matched control groups on stimulation with the virus (Figure 3). However, the level of IFN-α was significantly higher for Biobran/MGN-3-supplemented subjects relative to controls, which is in keeping with increased pDC activation.

IL-29 levels were also higher for the Biobran/MGN-3 group, but the differences were not significant. When we investigated other cytokines/chemokines, we observed that IP-10, MCP-1, and CCL-19 were all significantly induced in both groups on viral stimulation. In contrast, IL-6 was significantly induced in only the Biobran/MGN-3 group, not the controls. In keeping with the primed state of DCs at baseline, we observed significantly higher production of MCP-1, IL-6, and CCL-19 at baseline as compared to the age-matched control group (Figure 3). Taken together, these data indicate that Biobran/MGN-3 boosts/primes the innate cytokine/chemokine production at baseline and induces increased production of antiviral IFN-α after stimulation with SARS-CoV-2.

### 3.3. IFN-α Secretion at Day 7

**Figure 4:**
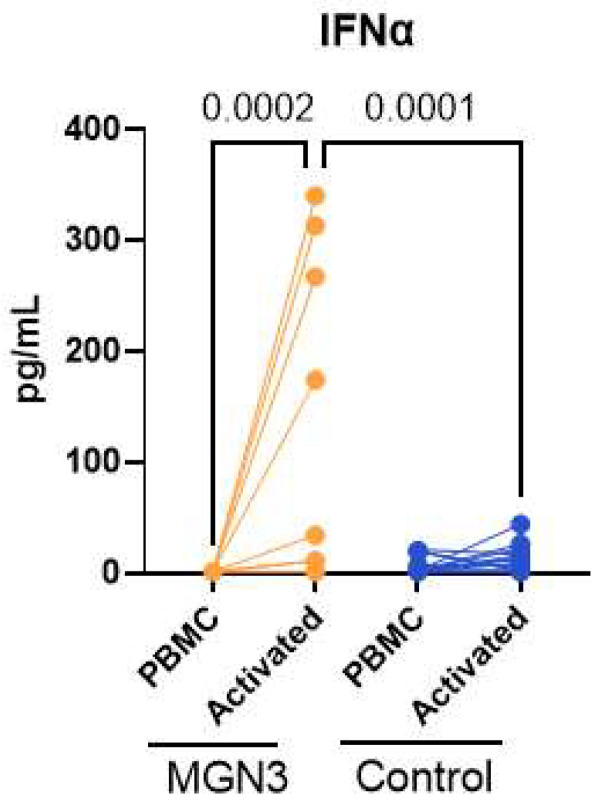
Quantitation of IFN-α in the supernatant at day 7. Lines connect the unactivated and activated conditions in the same subject. *N* = 8 for both Biobran/MGN-3-supplemented subjects and for age-matched controls. A Wilcoxon matched pairs signed-rank test was used to calculate *p*-values between the control and SARS-CoV-2-stimulated conditions in males and females. The unpaired *t*-test was used to calculate significance.

We also determined innate IFN-α secretion at day 7 after stimulation to determine whether the priming of DCs by Biobran/MGN-3 induces or prolongs the secretion of other cytokines. Most cytokines/chemokines tested returned to baseline. However, IFN-α secretion was significantly larger for the Biobran/MGN-3 group relative to controls (Figure 4), even at day 7. These data indicate that Biobran/MGN-3 sustains high levels of IFN-α on viral stimulation.

### 3.4. T Cell Responses in the Biobran/MGN-3-Supplemented Group

**Figure 5:**
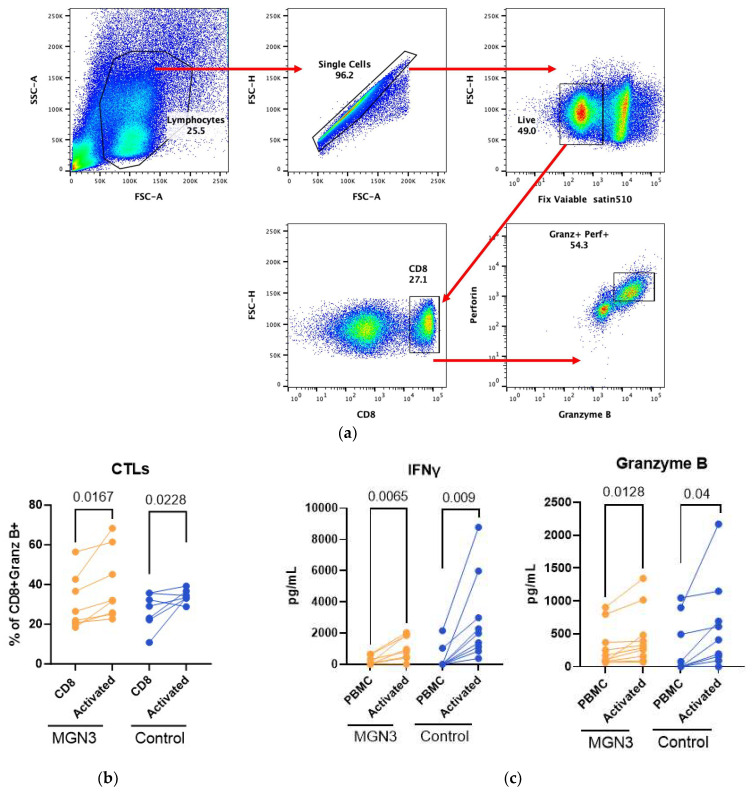
Cytotoxic T lymphocytes. PBMCs were stimulated for 7 days, and cytotoxic T cells (CTLs) were checked by flow cytometry. Cells collected were stained for CD8, perforin, and granzyme B. (**a**) Plots show the percentage of these cells obtained via flow cytometry. Gating strategy cells were stained with fixable live dead cells, and gated live cells were checked for singlet population. Further gated CD8 cells. Cells were fixed and permeabilized, and stains for Granzyme B and perforin were checked; (**b**) CTL generation on viral stimulation; (**c**) quantification of cytokines in the supernatants in Biobran/MGN-3 supplement-taking subjects (8 subjects) and age-matched controls (8 subjects). Lines connect the unstimulated and stimulated conditions in the same subject. The paired *t*-test (Mann–Whitney test) was used to calculate *p*-values.

Since priming of cytotoxic CD8 T cell responses by DCs and monocytes is essential to fighting viruses, we investigated this by determining the T cell responses 7 days after SARS-CoV-2 stimulation. Briefly, PBMCs were activated with the SARS-CoV-2 virus for 7 days and stained with viability stain 510. Figure 5a shows the gating strategy. CD8, intracellular granzyme B, and perforin were used to identify cytotoxic T cells (CTLs) (Figure 5b). Significant induction of CTLs was observed in both groups. The percentage of CTLs expressing granzyme B was higher in the Biobran/MGN-3 group both at baseline and after stimulation relative to the controls, though differences were not significant. The T cell cytokines IFN-γ and granzyme B were also induced significantly in both groups after viral stimulation. No significant difference was observed between groups (Figure 5c). These data thus indicate that the Biobran/MGN-3 and control groups both induce CD8 T cell responses.

## 4. Discussion

The COVID-19 pandemic has negatively impacted health across the globe for millions of people. It can cause serious or fatal illnesses, and it affects people of all ages. Populations such as the elderly are particularly susceptible to this infection [43,44] because immune responses decline with increasing age [45,46]. There is a need to enhance or boost the immune response in these populations. Biological response modifiers (BRMs) are a class of neutro-chemicals that have been found to activate the immune system; these can lead to enhanced capacity to fight against infections. BRMs may act on trained immunity, like the BCG and polio vaccines, which have been shown to reduce the incidence of COVID-19 [47,48].

Biobran/MGN-3 is a potent BRM that activates a wide range of immune cells, such as DCs, NK cells, and B cells [22]. We recently conducted a pioneering study showing that Biobran/MGN-3 exerts antiviral activity against SARS-CoV-2 in vitro [31]. The study explored Biobran/MGN-3′s therapeutic potential against coronaviruses through mechanisms involving the ability of its active ingredients to inhibit the coronavirus’s ability to enter host cells and replicate. The possible mechanisms underlying Biobran/MGN-3′s anti-SARS-CoV-2 effects were explored in silico via a study of serine carboxypeptidase and ferulic acid docking. Wheat bran and crude rice bran contain these two compounds, which are known to possess antioxidant, anti-inflammatory, and anticancer properties [49,50]. Specifically with regard to Biobran/MGN-3′s possible impact against SARS-CoV-2, we studied serine carboxypeptidase’s and ferulic acid’s binding to DC-SIGN and ACE2. Such bindings would interfere with SARS-CoV-2′s capabilities to enter cells and replicate.

The present study investigates whether healthy individuals taking Biobran/MGN-3 orally have better immune responses to SARS-CoV-2 relative to age-matched controls. Subjects taking Biobran/MGN-3 showed a markedly increased activation of pDCs on stimulation with SARS-CoV-2. Both mDCs and pDCs also expressed higher levels of HLA-DR at baseline as compared to controls, indicating that Biobran/MGN-3-treated subjects’ immune cells are already primed to fight infection. The secretion of IFN-α, the antiviral cytokine that helps eradicate viral infection [51,52], was also upregulated in Biobran/MGN-3-supplemented subjects. Furthermore, the secretion persisted for seven days after SARS-CoV-2 stimulation, indicating that the priming of DCs, particularly pDCs, by Biobran/MGN-3 supplementation not only enhances the secretion of IFN-α but also sustains it for a longer duration.

Biobran/MGN-3 supplementation also upregulated the chemokines CXCL-10, MCP-1, and CCL19 at baseline (Figure 3). CCL19 regulates the induction of T cell activation, inflammatory responses, and immune tolerance during continuous immune surveillance, homeostasis, and development [53]. The CXCL-10/CXCR3-A axis contributes to the proliferation of various cell types and the induction of chemotaxis [54], while the CXCL-10/CXCR3-B axis induces apoptosis but inhibits proliferation and migration [55,56]. CXCL-10 has also been shown to act as an antitumor/antiangiogenic protein [56]. CCL-2 is also a chemoattractant for macrophages. Elevated levels of these chemokines at homeostasis once again support Biobran/MGN-3′s priming of immunity.

Studies have found Biobran/MGN-3 to be effective against viral infection. Biobran/MGN-3 acts against HIV in vitro [38], and it can significantly lower the viral load of patients infected with chronic HCV [39]. Rat studies showed that Biobran/MGN-3 treatment significantly protects against D-GalN-induced liver injury and hepatitis [57], and Biobran/MGN-3 of low molecular fraction also protects against acute liver injury through the inhibition of JNK/MAPK and NF-кB expression [58]. Further work has also found that Biobran/MGN-3 consumption by elderly subjects reduced their incidence of influenza-like illnesses (ILI) [37]. As previously mentioned, Biobran/MGN-3 has furthermore been found to exert potent antiviral activity against the coronavirus, specifically against SARS-CoV-2, in vitro and in silico [31]. The present study demonstrates its possible anti-SARS-CoV-2 effects in human subjects in order to document its antiviral activity. Our results show that IFN-α levels are significantly higher relative to control subjects.

IFN-α has recently been considered as a possible therapeutic agent for treating the COVID-19 illness, primarily motivated by the fact that the innate immune system quickly produces IFN-α as an initial defense against viral infection. In addition, IFN-α is a key component in immunoregulatory and antiviral effects in COVID-19 patients [59]. Upon host infection, IFN-Is, particularly IFN-α and IFN-β, are produced and released into the innate immune system to combat viral infection [60]. Earlier studies showed that IFN-Is are some of the initial cytokines to be induced upon viral infection to mount the host’s immune response. This occurs when IFN-I binds to the IFN-α and IFN-β receptors on the cell surface, leading to transcription of interferon-stimulated genes (ISGs); these proteins then block SARS-CoV-2′s viral replication and spread [61].

Clinically, the use of IFN-α in COVID-19 patients has already been studied in connection with lopinavir/ritonavir (LPV/r). Patients injected subcutaneously with IFN-α2b combined with LPV/r were shown to have briefer lengths of hospitalization and a lower viral load relative to patients treated only with oral LPV/r [62]. Clinical studies in Cuba also found IFN-α2b to be effective against COVID-19 [63]. Recent studies suggest that COVID-19 severity may be related to the subtypes of IFN-α induction. IFN-α subtypes have been reported to be differentially induced in pathogenic influenza virus-infected human epithelial cells of mice, along with various degrees of inflammatory response [60].

NK cells are essential for initial defense against viral infections and cancer because of their ability to mediate spontaneous cell-mediated cytotoxicity for a range of virally infected cells and malignant tumors [64,65]. Earlier studies show that Biobran/MGN-3 is a potent BRM that enhances the activity of NK cells in different models. These include in vitro studies of cultured murine splenic lymphocytes and human PBLs from healthy individuals [66,67,68,69]. The immunomodulatory effects of Biobran/MGN-3 were also noted in vivo using aged mice and their associated decline in immune function. Biobran/MGN-3 treatment resulted in enhanced splenic and peritoneal NK activities, increased percentages of conjugates between NK cells and YAC-1 tumor cell targets, and increased the granular content of NK cells [68]. Further studies showed enhanced human NK cell activity after ingestion of Biobran/MGN-3, with oral administration significantly increasing NK activity in a dose-dependent manner [66]. Biobran/MGN-3 treatment also led to a notable increase in cancer patients’ NK cell activity [70,71]. This increase in activity did not significantly change the NK cell subsets (CD56+, CD16+) or the total NK cell population [66].

NK cells and CD8+ T cells identify and kill virally infected or transformed cells via the granular exocytosis pathway, i.e., they achieve their effect by delivering cytotoxic granules to target cells, leading quickly to death [72]. Biobran/MGN-3 treatment has been found in several studies to lead to increased granular content (perforin and granzyme B) in NK cells, as explored biochemically and morphologically [68,70]. The results clearly demonstrated that Biobran/MGN-3 treatment results in increased binding capacity of NK cells to their target cells. Biobran/MGN-3 also enhances the generation of cytotoxic CD8+ T cells by upregulating the expression of DEC-205 on DCs. Several reports have indicated the essential roles of DEC-205 and CD8+ T cell responses against viruses and cancer [73]. We recently demonstrated that stimulating DCs with Biobran/MGN-3 induces highly cytolytic CD8+ T cells and significant levels of granzyme B-positive CD8+ T cells. These results were linked with increased production of type III IFN and increased expression of DEC-205 [33]. Furthermore, Biobran/MGN-3-stimulated DCs induce CD4+ T cell proliferation and their production of cytokines. Its treatment has been found to stimulate DCs to induce the proliferation of CD4+ T cells and their production of the cytokines IFN-γ, IL-10, and IL-17 [32].

NK cells are critical mediators of antiviral immunity and can also regulate the broader immune response via cytokine production, such as for TNF and IFN-γ. Extensive studies have shown an association of depleted peripheral NK cells in patients with severe COVID-19 [74,75,76,77], and recent studies have characterized the response of NK cells to COVID-19 [78]. Reasons for NK cell dysfunction in COVID-19 patients were attributed to the significant reduction in cytokine induction and surface expression of key activating receptors, as well as the increased expression of the inhibitory receptor NKG2A. All of these factors led to the overall suppression of the NK cells’ ability to form conjugates with their target cells [78]. In contrast, we have demonstrated that Biobran/MGN-3 is a potent BRM capable of significantly enhancing NK cell activity in animals and humans [36,66,68]. Therefore, we believe that the apparent anti-COVID-19 effects in the present study may be due to the enhancement of NK cell activity by Biobran/MGN-3.

This study has a few limitations. The dosage of Biobran/MGN-3 treatment varies between 1 and 2 gm/day, and the length of treatment is also not exact. In studying the mechanisms of action, Biobran/MGN-3 is known to be a potent antioxidant—the current study does not explore the overlap between the antioxidant and antiviral activities, but such a study would be very desirable to explore in the future. Furthermore, this study is not an in vivo study. We are studying the activation of dendritic cells and monocytes where the SARS-CoV-2 virus does not multiply. Our study with the irradiated virus is relevant to in vitro studies, and in vivo, the virus will further multiply in epithelial and other cells. The DCs and monocytes will still sense the virus in the same way as for the irradiated virus, but the response will also be affected by the factors secreted by the infected epithelial and other cells. Nevertheless, despite these limitations, this study provides key information regarding the effect of long-term supplementation of Biobran/MGN-3 on the human immune response to SARS-CoV-2.

## 5. Conclusions

Our data indicate that Biobran/MGN-3 supplementation induces increased pDC activation and IFN-α secretion on SARS-CoV-2 stimulation. The immune system is also primed by Biobran/MGN-3, as evident by increased activation of pDCs and mDCs at baseline and secretion of chemokines. These data thus support the use of Biobran/MGN-3 as a potential immune booster against SARS-CoV-2 and other viral infections in vulnerable populations.

## Data Availability

The data presented in this study are available on request from the corresponding author. The data are not publicly available due to privacy.
